# The Curious Case of Lip Tongue Fusion: A Consequence of Suboptimal Oral Care

**DOI:** 10.1155/2022/3113886

**Published:** 2022-11-08

**Authors:** Jimmy Xu, Prateek Biyani, Andrew Rajkumar, Robert Orr

**Affiliations:** ^1^Royal Derby Hospital, Derby, UK; ^2^Chesterfield Royal Hospital, Chesterfield, UK

## Abstract

Oral care is an often difficult and an unappreciated part of hospital life. Patients who are unable to provide their own care rely on assistance from hospital personnel. Most sequelae from suboptimal oral care often present over months if not years, in the form of dental caries and periodontal disease. We present an exception, where a 66-year-old patient who experienced widespread ulceration and necrosis from *Capnocytophaga*-related sepsis received suboptimal oral care, resulting in their tongue being fused to their lip. This was later divided by the oral and maxillofacial team resulting in restoration of full function. Future cases can be avoided in patients with similar symptoms, such as Stevens-Johnson syndrome or erythema multiforme, if rigorous oral care can be provided.

## 1. Introduction

Good mouth care in an intensive care setting is crucial; however, unfortunately, it is often carried out in a suboptimal manner. This is understandable due to patients being more complicated to manage, with multiple other morbidities including confusion and lack of cooperation. We present a case of a patient whose tongue, as a result of suboptimal oral care, has granulated onto their lip.

## 2. Case Presentation

A 66-year-old male attended a neighbouring accident and emergency department with an unknown cause of sepsis and multiorgan failure. The patient had no relevant medical history apart from asplenia as a result of splenectomy after a previous postfundoplication complication. He was socially independent.

Initially presenting to a neighbouring hospital due to shortness of breath, fever, confusion, and feeling unwell for 24 hours, our patient was admitted under a medical ward briefly before starting to rapidly desaturate on room air and oxygen. A CT chest-abdomen-pelvis showed bilateral mild pleural effusion with a mildly dilated common bile duct, and a CT head showed no abnormalities. Blood, sputum, urine, and bronchoalveolar lavage cultures proved negative, and meropenem was started empirically. He later developed septic cardiomyopathy with an ejection fraction of 10%, and later, complete anuria. The patient had developed multiorgan failure and was eventually mechanically ventilated via an endotracheal tube at the neighbouring hospital.

The patient was later transferred to the intensive care unit at Chesterfield Royal Hospital five days after initial presentation where he was placed on norepinephrine, continuous venovenous hemofiltration, and continued on continuous positive airway pressure. A tracheostomy was later provided and sedation weaned off when symptoms started to improve. Upon presentation at our hospital, the skin to the majority of his body was extremely mottled, bleeding, and blistered, with severe purpura and peripheral ischemia, especially peripherally. This had apparently developed 2 days before transfer. The mottled purple appearance of his skin gave the initial impression of vasculitis secondary to infection; however, the presence of blisters was unusual for this as it was also on undamaged skin. Both haematological screening and skin biopsies later proved negative for vasculitis. After a subsequent positive blood culture, it was discovered that this patient had *Capnocytophaga*-related fulminant sepsis, a rare complication arising from a calf wound licked by his dog several days before initial presentation. Microbiology had recommended both Tazocin and Teicoplanin based on sensitivity. As the patient's symptoms were already improving, antibiotics had already been stopped a week prior.

**Figure 1:**
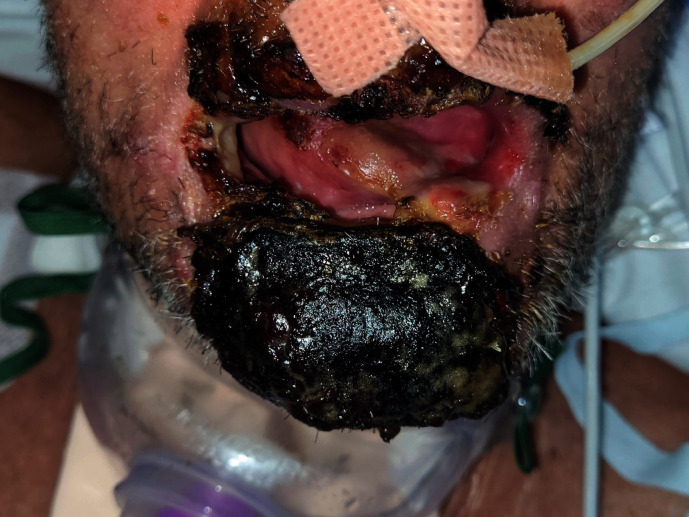
Tongue tethered to lower left lip due to granulation and associated extensive necrosis.

**Figure 2:**
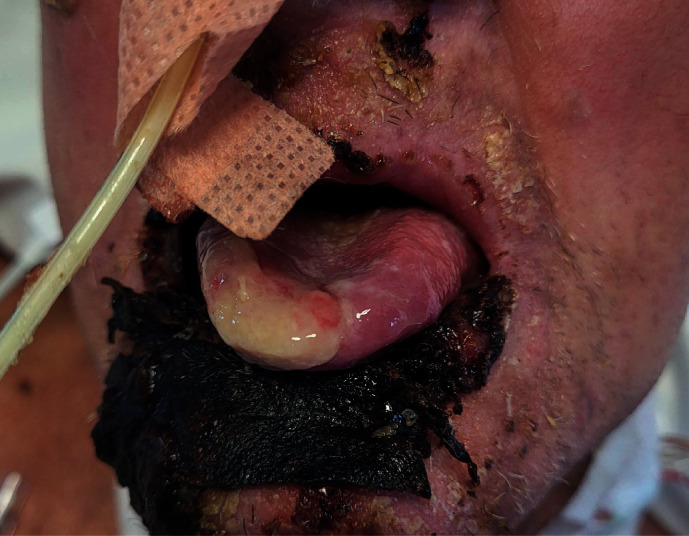
Tongue after division of fused tissues with full range of movement.

The normal mouth care regimen at our hospital involves cleaning all intraoral surfaces with a solution of 0.2% chlorhexidine using a soft brush approximately every 4 hourly and to brush teeth twice daily for those in critical care. Unfortunately, mouth care was not always performed intraorally due to the patient not being able to open his mouth when asked to. Additionally, staff were hesitant to clean intraorally whilst the patient was intubated due to the severity of the necrosis, resulting in bleeding when attempted. Intraoral care was therefore done very reluctantly, if at all. Our oral and maxillofacial surgery department was later involved when speech and language therapy noted the patient's tongue tip was fused to the left side of his lip resulting in impaired swallowing. They had initially used a toothbrush to lightly rub a pale coating between the tongue and the lip as it was thought to be dried secretions, but instead the area started to bleed, leading to the finding. This was discovered just under 3 weeks after admission, but approximately 2 weeks after oral blistering started. Upon further examination by our team, the tongue appeared to have granulated into the patient's lip as a result of the generalised necrosis in the oral cavity, along with previous lack of tongue movement (Figure 1). The attachment was later carefully divided under local anaesthetic using 2% lidocaine with 1 : 80000 adrenaline and a 15 blade without any complications (Figure 2). The patient regained full range of movement and restoration of function immediately after. Unfortunately, the patient started deteriorating approximately a week later, also acquiring pseudomonas and coagulase negative staphylococci bacteraemia, and has now passed away.

## 3. Discussion

This case not only demonstrates the difficulty of managing *Capnocytophaga*-related sepsis, but also the significant importance of good oral care in intensive care patients.

*Capnocytophaga spp* are an opportunistic genus of gram-negative bacteria which can cause a varied presentation of infections [1]. *Capnocytophaga canimorsus*, specifically, can be transferred to humans via saliva in dogs or cats, especially in open wounds. Immunocompromised patients are therefore at a high risk of developing serious complications such as sepsis, meningitis, and respiratory distress from this condition [2]. Patients who have had their spleens removed are therefore more prone to serious infections including to *Capnocytophaga* and appear to develop more serious symptoms than those who are splenic [3–5]. Other at risk groups include patients with diabetes, liver cirrhosis, and those with a history of alcohol abuse [6].

The mainstay of treatment for *Capnocytophaga*-related sepsis are antibiotics. The current consensus appears to be penicillins with beta-lactamase inhibitors, due to increasing incidence of penicillin resistant strains [7]. Patients with dermatological symptoms, such as purpura fulminans or thrombocytopenia purpura, appear to not be uncommon [8], and this can evidently have an impact on mouth care if present in the perioral and intraoral tissues.

In the intensive care setting, cleaning the mouth is recognised as especially important for plaque control, and potentially reducing systemic infection [9]. Furthermore, the reduction in bacterial load and disrupting the biofilm is critical in reducing dental caries [10] and periodontal disease for those individuals unable to take care of themselves. Despite this, the priority is often low amongst staff, especially with uncooperative and confused patients [11].

There are no current reports in the literature of such extensive ulceration and necrosis on the mucosal tissues causing fusion intraoral tissues. The combination of severe generalised ulceration along with immobility to intraoral tissues for at least several days appears to be an exceptionally rare occurrence. Nonetheless, our case report helps to demonstrate that such an event can occur and highlights one of the rare complications of not only *Capnocytophaga*-related sepsis but also other conditions which could result in widespread necrosis and ulceration, such as Stevens-Johnson syndrome and erythema multiforme. This situation could have ultimately been avoided through more rigorous oral care practices.
